# Quick and Cheap: Optimized Purification and Concentration of Bacteriophages Produced in Rich Culture Media

**DOI:** 10.21769/BioProtoc.5608

**Published:** 2026-02-20

**Authors:** Amel Chaïb, Laura Schmitt, Adeline Goulet, Claire Le-Marrec

**Affiliations:** 1 Univ. Bordeaux, INRAE, Bordeaux INP, UMR 1366, OENO, ISVV, Villenave d’Ornon, France; 2 Laboratoire d’Ingénierie des Systèmes Macromoléculaires (LISM, UMR7255), Institut de Microbiologie de la Méditerranée, Aix Marseille Université, Centre National de la Recherche Scientifique, Marseille, France

**Keywords:** Phage purification, Phage concentration, Dialysis, *Oenococcus oeni*, Negative stain electron microscopy

## Abstract

This protocol describes an easy, quick, cheap, and effective method for the purification and concentration of bacteriophages (phages) produced in rich culture media, meeting the quality criteria required for structural analyses. It is based on a tube dialysis system that replaces the classical but expensive and tedious density gradient ultracentrifugation step. We developed this protocol for the *Oenococcus oeni* bacteriophage OE33PA from its amplification to imaging by negative stain electron microscopy (NS-EM). The host bacterium, *O. oeni*, is a lactic acid bacterium that lives in harsh oenological ecosystems and grows only in rich and complex media such as Man–Rogosa–Sharpe (MRS) or fruit juice-based media in laboratory conditions. This raises experimental challenges in pure and concentrated phage preparations for further uses such as structure-function studies.

Key features

• Simple, rapid, and cheap, this method provides a fast and easy approach for efficient bacteriophage purification and concentration.

• The method meets the structural study requirements of phage particles.

• The method is compatible with bacteria that grow only in complex and rich culture media.

• This protocol is also compatible with the purification of phages that produce low-titer lysates.

## Graphical overview


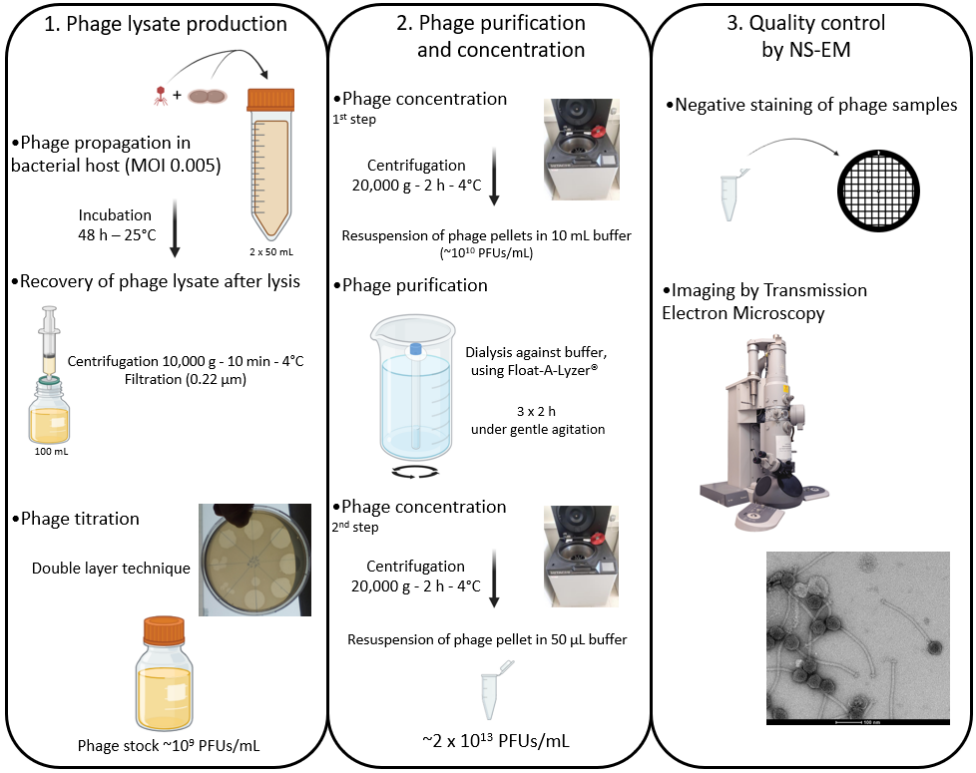


**Flowchart illustrating the production, purification, and concentration protocols of phages produced in rich culture media before observation by negative stain electron microscopy (NS-EM).** MOI, Multiplicity of infection; PFU, plaque forming unit.

## Background

Bacteriophages (phages, viruses of bacteria) are major players in all ecosystems, whether they are natural or anthropic [1]. They affect their bacterial hosts in different ways: they regulate bacterial populations via predation, improve their host fitness (i.e., lysogenic conversion), and are drivers of bacterial evolution [2]. Moreover, given their potential to be used as molecular tools [3], they are increasingly studied in a wide range of disciplines, from medicine to ecology or biotechnology.

Phages, like all viruses, have a “nanoscopic” size and are unable to propagate on their own. However, they can be seen by transmission electron microscopy (TEM), and their propagation can be followed through cell lysis of their host. It is worth pointing out that the existence of phages, as biological entities, was definitively proven by TEM imaging more than 20 years after their discovery [4].

The description of virion structure is part of the basic characterization of phages. Yet, it is not uncommon to come across poor-quality TEM micrographs in the literature, showing phage preparations contaminated by components of the bacterial culture medium. In most studies, bacteriophages are purified using cesium chloride (CsCl) density gradient ultracentrifugation, which separates particles by buoyant density [5]. While this method yields highly pure phage preparations, it is labor-intensive, requires expensive ultracentrifugation equipment, and involves CsCl, which is toxic for operators and poses handling risks. To avoid using this type of gradient, we previously developed a simple protocol for the preparation of the GC1 phage amplified upon infection of its host, the acetic-acid bacterium *Gluconobacter cerinus* in poor media, which is efficient enough to observe virion ultrastructure using cryo-electron microscopy (cryo-EM) [6,7]. Here, we present an improved protocol for the preparation of phages whose hosts grow in complex and rich media based on the use of a tube dialysis system.

This protocol was developed using the *Oenococcus oeni* phage OE33PA, an ex-temperate phage (i.e., a mutant only able to perform lytic infections) previously isolated from wine [8]. The host, *O. oeni*, has a fastidious growth that occurs only in rich culture media such as Man–Rogosa–Sharpe (MRS) or fruit juice-based media, which makes the purification of its phages complicated for later uses, such as structural analyses. In the present article, we show that our optimized protocol is simpler and more efficient than the one we published in 2020 [7] to produce high-quality GC1 phage particles.

## Materials and reagents

**Biological materials**

1. *Oenococcus oeni* S277 [9]

2. OE33PA phage suspension [8]

**Reagents**

1. Tris-HCl pH 7.5 (Fisher Scientific, catalog number: 10123722)

2. Sodium chloride (NaCl) (Fisher Scientific, catalog number: 10055850)

3. Magnesium sulfate heptahydrate (MgSO_4_·7H_2_O) (Fisher Scientific, catalog number: 10135453)

4. Calcium chloride dihydrate (CaCl_2_·2H_2_O) (Fisher Scientific, catalog number: 10158280)

5. Hydrochloric acid (HCl), 33% (w/v) aqueous solution (Fisher Scientific, catalog number: 11331588)

6. Granulated agar (Dutscher, catalog number: 214510)

*Note: All above chemicals, which are used in media recipes, are reagent grade (ACS grade).*

7. Uranyl acetate dihydrate 98% (Sigma-Aldrich, withdrawn from the market)

8. MRS (Difco, BD, Fisher Scientific, catalog number: 13689811713553)

**Solutions**

1. Man–Rogosa–Sharpe (MRS) broth (see Recipes)

2. MRS and MRSΦ solid medium (see Recipes)

3. MRSΦ soft agar medium (see Recipes)

4. 10× phage buffer (ΦB) (see Recipes)

**Recipes**

**1. MRS broth**

**Table:** 

Reagent	Final concentration	Quantity or volume
MRS powder	55 g/L	55 g

a. Make up the volume to 1 L with distilled water.

b. Adjust the pH to pH 4.8 with HCl 33.33% (w/v) aqueous solution.

c. When needed, add 1.77 g of CaCl_2_·2H_2_O and 1.8 g of MgSO_4_·7H_2_O (e.g., for MRSΦ, used for phage propagation).

d. Sterilize by autoclaving.

e. Store at 4 °C for up to 6 months.

**2. MRS and MRSΦ solid medium**

**Table:** 

Reagent	Final concentration	Quantity or volume
MRS powder	55 g/L	55 g
Granulated agar	20 g/L	20 g

a. Make up the volume to 1 L with distilled water.

b. Adjust the pH to pH 4.8 with HCl 33.33% (w/v) aqueous solution.

c. Sterilize by autoclaving.

d. Store at 4 °C for up to 6 months.

**3. MRSΦ soft agar medium**

**Table:** 

Reagent	Final concentration	Quantity or volume
MRS powder	55 g/L	55 g
Granulated agar	6 g/L	20 g
CaCl_2_·2H_2_O	16 mM	1.77 g
MgSO_4_·7H_2_O	15 mM	1.8 g

a. Make up the volume to 1 L with distilled water.

b. Dissolve agar by heating.

c. Aliquot 5 mL of the medium in approximately 200 glass tubes (20 mL).

d. Sterilize by autoclaving.

e. Store at 4 °C for up to 6 months.

**4. Phage buffer 10**× **(ΦB)**

**Table:** 

Reagent	Final concentration	Quantity or volume
NaCl	1 M	58 g
MgSO_4_·7H_2_O	0.08 M	20 g
1 M Tris-HCl pH 7.5	0.5 M	500 mL
Distilled water	n/a	500 mL
Total	n/a	1000 mL

a. Sterilize by autoclaving.

b. Store at room temperature for up to 6 months.

c. To prepare 1× ΦB, dilute 10 mL of 10× ΦB with 90 mL of sterile distilled water.

**Laboratory supplies**

1. 50 mL centrifuge tubes (Dutscher, catalog number: 352070)

2. 100 mL SHOTT bottle (Dutscher, catalog number: 090441A)

3. 250 mL SHOTT bottle (Dutscher, catalog number: 090346)

4. 1.6 mL spectrophotometer cuvettes (Dutscher, catalog number: 030101)

5. 0.22 μm PES membrane syringe filters (Fisher Scientific, catalog number: SLGP033NK)

6. 50 mL sterile disposable plastic syringes (Fisher Scientific, catalog number: 10119350)

7. 1.5 mL polypropylene microcentrifuge tubes (Fisher Scientific, catalog number: 10154671)

8. Sterile 90 × 13 mm Petri dishes (Dutscher, catalog number: 076084E)

9. 20 mL autoclavable glass tubes (Dutscher, catalog number: 045209)

10. Float-A-Lyzer^®^ G2 100 kDa 10 mL (Fisher Scientific, catalog number: 11561170)

11. 300 mesh 3 mm carbon-coated copper grids (TAAB, catalog number: C267/100)

12. Whatman filter paper grade 5 (Dutscher, catalog number: 1005055)

## Equipment

1. Spectrophotometer (Shimadzu, model: UV-1280)

2. Incubator set to 25 °C

3. Water bath set to 55 °C

4. High-speed refrigerated centrifuge (Hitachi, model: CR 22N) and R15A-0688 rotor

5. pH meter (Hanna, model: pH211)

6. Magnetic stirrer

7. Glow discharge unit (Electron Microscopy Sciences, model: GloQube Plus)

8. Transmission electron microscope (Tecnai, model: G2 Spirit T12 TEM)

9. CCD camera (Olympus, Germany, model: Veleta 2k × 2k Side-Mounted TEM CCD Camera Solution)

## Procedure

**A. Production and titration of OE33PA lysates**

1. Preparation of a S277 sensitive host pre-culture

a. In a sterile 50 mL tube, introduce aseptically 50 mL of MRS broth.

b. Inoculate approximately 100 μL of an S277 frozen culture.

c. Incubate over 5 days at 25 °C statically.

2. S277 infection by OE33PA phage and recovery of the phage lysate

a. Measure the OD_600_ of the S277 pre-culture to prepare 150 mL cultures at an OD_600_ of 0.1 (corresponding to approximately 10^8^ CFUs/mL) in a sterile 250 mL bottle. Use MRSΦ broth.

b. Divide the pre-culture into three separate 50 mL sterile tubes.

c. Introduce a volume of OE33PA phage suspension in two of the three cultures to obtain a high multiplicity of infection (MOI) of 1 phage for 200 bacteria. The third culture is used as an uninfected negative control for lysis. For example, if the initial phage titer is 10^9^ PFUs/mL, then 25 μL must be added to 50 mL of bacterial suspension.

d. Incubate for 48 h at 25 °C statically.

e. Note the lysis of the infected cultures by comparing their OD_600 _with that of the control. Measurements are generally around 0.05 for the infected cultures and around 1 for the control.

f. Centrifuge the lysed cultures at 10,000× *g* for 10 min at 10 °C.

g. Transfer the supernatants to a 50 mL syringe and filter through a 0.22 μm PES membrane in a sterile 100 mL bottle. Pool the two 50 mL lysates in the same bottle.

*Note: The host bacterium S277 needs to be in the exponential growth phase for efficient phage propagation and enumeration.*

**Figure 1.**
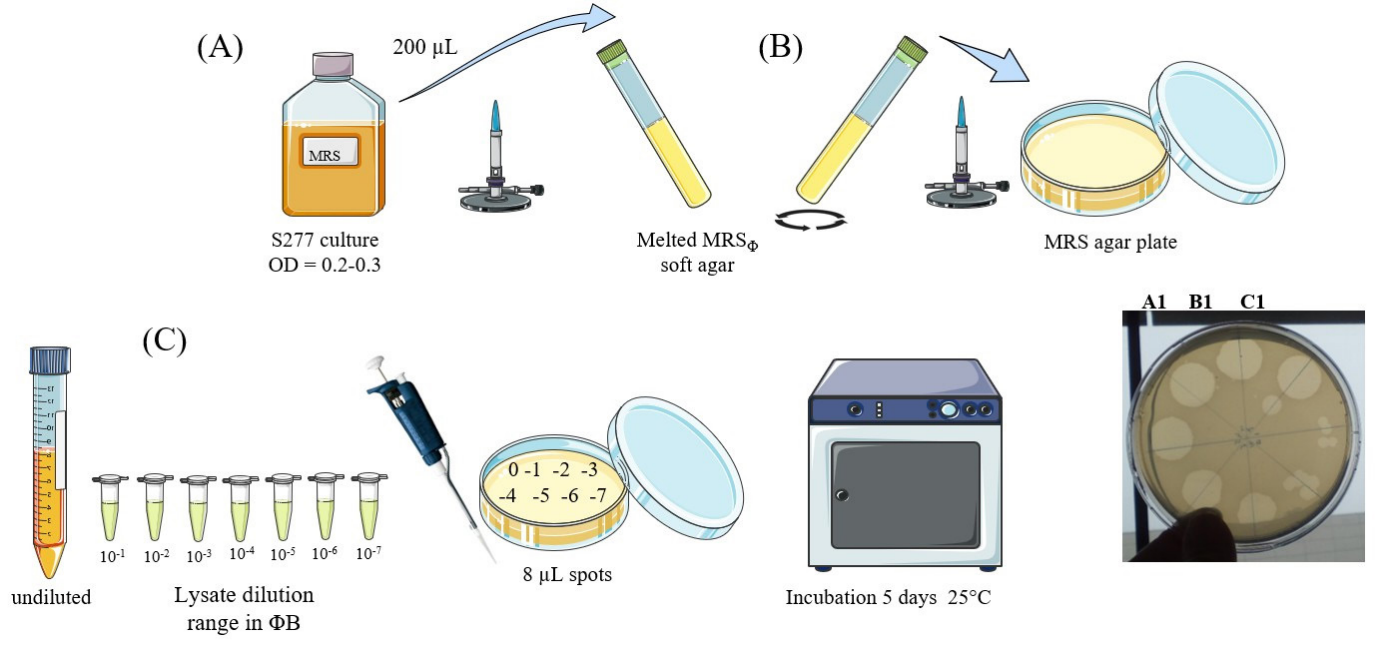
Rapid titration of phage lysate. (A) Bacterial culture preparation. (B) Bacterial lawn in soft agar medium preparation. (C) Phage titration.

3. Rapid titration of OE33PA lysate through the double-layer method (Figure 1)

a. Prepare a ten-fold serial dilution of the lysate to 10^-7^ in 1 mL of phage buffer (ΦB) 1× using 1.5 mL microcentrifuge tubes.

*Note: Dilutions can be stored for a few days at 4 °C.*

b. Bring two tubes containing 5 mL of MRSΦ soft agar to supercooling by heating. Cool down to approximately 55 °C in a water bath.

c. Take two solid MRS plates and divide one of the plates into 8 equal quadrants.

d. Prepare 1 mL of S277 fresh culture at an OD_600_ of 0.2 in a microcentrifuge tube.

e. Inoculate 0.2 mL of the S277 culture in each MRSΦ soft agar tube.

f. Homogenize and pour a tube of inoculated soft agar on each plate of MRS and let it solidify. This step must be carried out quickly so that the agar remains supercooled.

g. Apply a 0.008 mL drop of each dilution (undiluted to 10^-7^) on a square of the quadrated plate. Let air dry under sterile conditions. The second plate is used as an uninfected negative control.

h. Incubate for 4–5 days at 25 °C.

i. Count the isolated lysis plaque-forming units (PFU) at the lowest dilution and deduce the phage titer of the lysate using the following formula:

$$N=\frac{\sum P}{v}d$$

where N, phage titer (PFUs/mL); P, number of counted PFUs; v, volume of the applied drop of phage suspension (mL); d, retained dilution factor.

*Note: OE33PA lysates often reach 10^9^ PFUs/mL.*

**B. Concentration and purification of phage particles**

1. Concentration of OE33PA phage particles

a. Centrifuge all remaining lysate volume in two sterile 50 mL centrifuge tubes at 20,000× *g* for 2 h at 4 °C. Indicate the anticipated localization of the pellets on the tubes.

b. Carefully remove the supernatant and dry the pellets.

c. Resuspend the phage pellets gently and aseptically in 5 mL of sterile ΦB 1× each.

*Note: The suspension can be stored at 4 °C for a few days. To be effective, the phage lysate must have a concentration of at least 10^9^ PFU/mL; otherwise, see step B3.*

2. Purification of phage particles

a. Fill a recipient with 1.2 L of 1× ΦB. Place the recipient on a magnetic stirrer and insert a rod magnet into the buffer.

*Note: Use a recipient high enough to allow agitation without the risk of colliding with the Float-A-lyzer^®^, for example, a 2 L beaker. The buffer does not need to be kept under aseptic conditions.*

**Figure 2.**
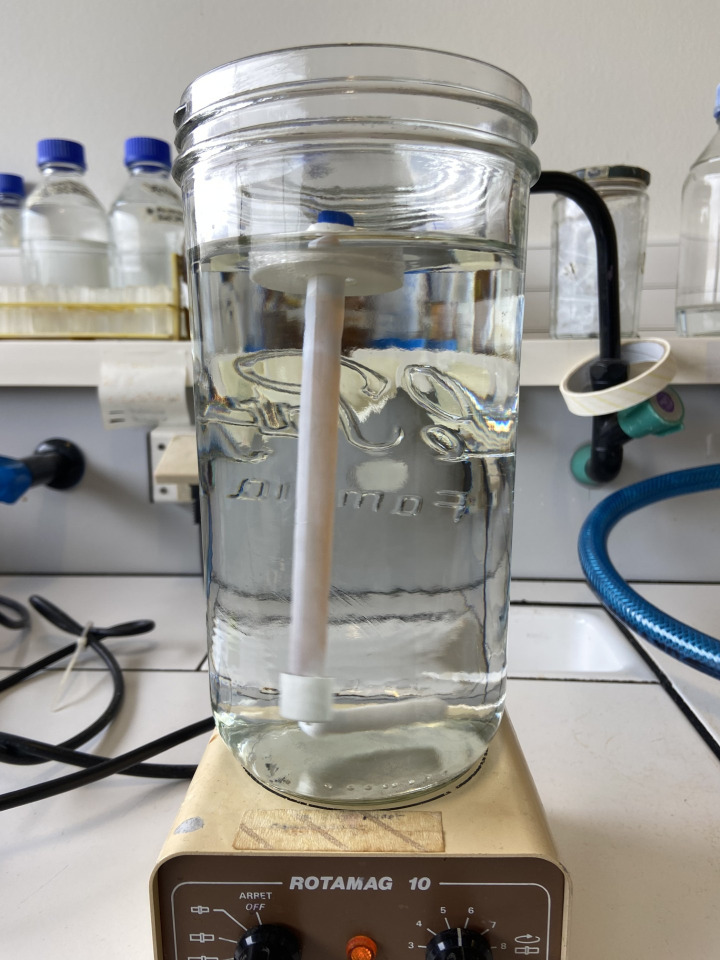
Tube dialysis system (Float-A-Lyzer^®^)

b. Transfer the two 5 mL phage suspensions aseptically into the Float-A-lyzer^®^ and close it with its screw cap.

c. Enclose the Float-A-lyzer^®^ tightly in its float and place it in 1× ΦB with gentle agitation. Leave to agitate for 1.5–2 h at room temperature (Figure 2).

d. Renew the buffer every 1.5–2 h as many times as necessary to ensure that the phage suspension is completely colorless. In general, two renewals are sufficient for efficient dialysis.

e. Transfer the phage suspension aseptically into a new 50 mL centrifuge tube.

f. Centrifuge at 20,000× *g* for 2 h at 4 °C. Indicate the anticipated localization of the pellet.

g. Carefully remove the supernatant and let the pellet air dry while maintaining the tube horizontally.

h. Resuspend aseptically the pellet into the desired volume of ΦB 1× (10–50 μL), depending on the desired phage concentration.

i. Store at 4 °C for a few weeks.

3. Alternative protocol for lysate with titers below 10^9^ PFUs/mL

*Note: Some phages do not give high titers, e.g., some temperate phages. In this case, a larger volume of lysate must be produced to obtain a sufficient number of phages. If, for example, the phage reaches 5 × 10^7^ PFU/mL, then 2,000 mL of lysate is required.*

a. Perform centrifugation as in step B1a–b, adapting it to the volume to be processed.

b. Resuspend gently and aseptically the phage pellet(s) in sterile ΦB 1×. If there are several pellets, use an adequate volume to obtain 10 mL of total phage suspension when pooled.

c. Continue the protocol from step B2a.

*Note: For electron microscopy (EM), 100 mL of lysate at a concentration of 10^7^ PFUs/mL is sufficient, but not for cryo-EM.*

**C. NS-EM imaging of phage preparations**

1. Preparation and imaging of negatively stained EM grids

a. Glow discharge 300 mesh carbon-coated copper grids with the carbon-coated side facing up for 1 min at 30 mA.

b. Apply 6 μL of the phage sample on the carbon-coated side of the grid for 30 s and remove the excess of sample by side-blotting with a piece of filter paper.

c. Briefly apply the grid on a 10-μL droplet of 1% uranyl acetate and blot the excess of liquid; then, stain the grid with a 10 μL droplet of 1% uranyl acetate for 10 s and blot dry.

d. Load the grid on a TEM operating at 120 kV and perform direct alignments.

e. Acquire images using a CCD camera, with an electron dose of 20e-/Å^2 ^at the desired magnification.

## Validation of protocol

**Figure 3.**
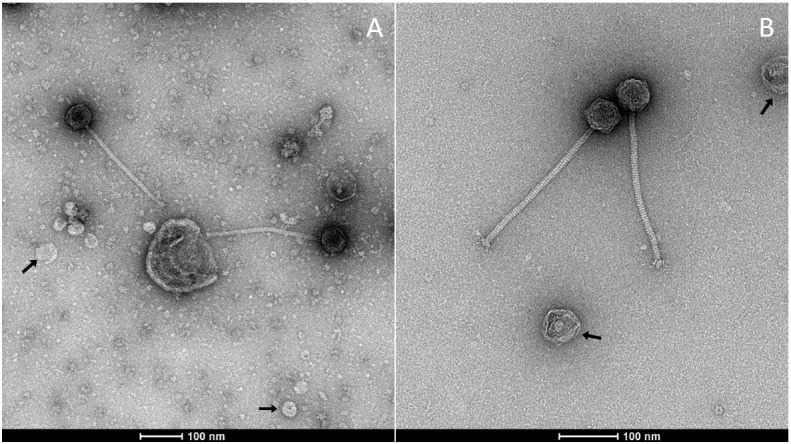
Observation of OE33PA viral particles prepared using different protocols. (A) Negative stain electron microscopy (NS-EM) micrograph of OE33PA prepared as in Chaïb et al. [7]. The presence of small-sized contaminants is not desirable for structural analyses; (B) NS-EM of OE33PA prepared with the present protocol. Of note, in both cases, we observe vesicles indicated by arrows that may be *O. oeni* extracellular vesicles.

To validate our optimized phage sample preparation protocol, we compared negatively stained (NS) electron micrographs of OE33PA obtained following the procedure described by Chaïb et al. [7] with those prepared using the present protocol (Figure 3). In the micrographs obtained with the previous method (A), small-sized contaminants are clearly visible, which are undesirable for structural analyses and may interfere with data interpretation. In contrast, samples prepared according to the current protocol (B) exhibit a markedly improved purity, with a significant reduction of such contaminants. Moreover, with the new protocol, the phage tail ultrastructure can be clearly distinguished, particularly the phage baseplate previously predicted using AlphaFold2 [10]. In both preparations, we note the presence of vesicular structures that could correspond to *Oenococcus oeni* extracellular vesicles, as previously reported [11]. This comparison confirms the efficiency and reliability of the new protocol for obtaining high-quality samples suitable for further structural characterization.

## General notes and troubleshooting

The head of phage OE33PA measures approximately 55 nm in diameter, so a molecular weight cutoff of 100 kDa, corresponding to pores of approximately 10 nm in diameter, was chosen. This size retains phages while allowing smaller molecules, such as metabolites, sugars, or endolysins, which generally have a size ranging from 25 to 40 kDa, to pass through the membrane [12]. In theory, this method can be used with any type of virus of similar size, whether tailed or not. For viruses of different sizes, the molecular weight cutoff must be adjusted.

The Float-A-Lyzer^®^ is easy to handle, but it must be used with care to prevent damage to the membrane. As long as the membrane remains intact, no phages are lost into the ΦB (data not shown).

According to the principle of osmolarity that governs dialysis, the buffer should be replaced once it takes on the same color as the phage suspension inside the Float-A-Lyzer^®^, which represents 1.5–2 h for the indicated volume. Changing it earlier does not impact the phage preparation. What ultimately matters is that, by the end of the dialysis process, both the phage suspension and the external buffer are completely colorless.
